# Melt Electrowriting High Resolution Poly(ethylene‐co‐vinyl acetate) Scaffolds for Soft Tissue Engineering

**DOI:** 10.1002/adhm.202504945

**Published:** 2026-02-24

**Authors:** Finn Snow, Darcy De Rauch, Lilith Mabel Caballero Aguilar, Darcy Millett, Jasley Wilding McBride, David R Nisbet, Magdalena Kita, Elena Pirogova, Robert Michail Ivan Kapsa, Anita Quigley

**Affiliations:** ^1^ Department of Biomedical Engineering School of Engineering RMIT University Melbourne Victoria Australia; ^2^ Aikenhead Centre for Medical Discovery St Vincent's Hospital Melbourne Victoria Australia; ^3^ The Graeme Clark Institute The University of Melbourne Melbourne Victoria Australia; ^4^ Department of Biomedical Engineering Faculty of Engineering and Information Technology The University of Melbourne Melbourne Victoria Australia; ^5^ Department of Medicine The University of Melbourne Melbourne Victoria Australia; ^6^ Melbourne Medical School Faculty of Medicine, Dentistry and Health Science The University of Melbourne Melbourne Victoria Australia; ^7^ Department of Clinical Neurosciences St Vincent's Hospital Melbourne Melbourne Victoria Australia

**Keywords:** biomaterials, melt electrowriting, polyethylene vinyl acetate, scaffolds, skeletal muscle, tissue engineering

## Abstract

Melt electrowriting (MEW) holds tremendous potential to advance regenerative engineering, yet its clinical translation is hindered by the inability to replicate the biomechanics of soft tissues. We present, for the first time, MEW of polyethylene vinyl acetate (PEVA), demonstrating its potential as a highly compliant and biocompatible polymer for high‐resolution scaffold fabrication. Optimized printing parameters displayed a highly stable jet, enabling microscale fibers to be fabricated with pore sizes down to 100 µm, achieving the highest diameter‐to‐spacing ratio reported to date for elastic MEW polymers. MEW PEVA fibers exhibited markedly enhanced mechanical compliance under tensile loading, with 4‐fold and 35‐fold greater compliance than thermoplastic polyurethane (TPU) and polycaprolactone (PCL), respectively, while maintaining yield strains comparable to TPU. Under compression, macroporous PEVA scaffolds showed an extended toe region of up to 75%, approximately 250‐fold greater compliance than PCL, and yield strains exceeding 85%. PEVA scaffolds supported strong cell attachment and survival, with initial growth dynamics analogous to PCL and a significant increase in metabolic activity by day 7. This breakthrough establishes PEVA as a transformative material that overcomes the mechanical mismatch between MEW scaffolds and native soft tissues, expanding its translational potential for soft tissue engineering.

## Graphical abstract

This work pioneers melt electrowriting (MEW) of polyethylene vinyl acetate (PEVA) to fabricate ultra‐compliant, high‐resolution scaffolds. By integrating microscale precision with soft tissue‐like biomechanics, PEVA overcomes stiffness‐driven limitations of conventional MEW polymers, establishing a mechanically biomimetic platform for soft tissue engineering.

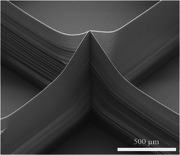

## 1 Introduction

Melt electrowriting (MEW) is an emerging additive manufacturing technique where molten thermoplastic polymers are drawn via electrohydrodynamic (EHD) forces to fabricate microscale, highly organized fibrous scaffolds. In contrast to solution electrospinning, MEW allows precise placement of filaments into predetermined architectures, typically with micrometer diameters [1, 2, 3]. This level of control makes MEW especially attractive for applications in tissue engineering, where scaffold geometry plays a critical role in dictating mechanical properties and directing cellular organization, differentiation, and tissue morphogenesis [4].

MEW continues to demonstrate remarkable utility in producing scaffolds that recapitulate key features of native extracellular matrix, including anisotropy, porosity, and mechanical integrity. Due to the ubiquitous use of the ‘gold standard’ PCL, however, advances are largely confined to hard tissue applications [1, 2, 3, 4, 5]. This stems from the critical need for implantable scaffolds to be mechanically similar to the targeted native tissue, ensuring biomechanics are maintained throughout tissue regeneration [6]. With mismatched mechanical properties, an implant's structural integrity may be unable to withstand applied loads, failing under physiological conditions. Conversely, overly rigid scaffolds absorb physiological loads, depriving mechanosensitive cells of essential mechanical cues required for tissue maturation, a concept known as stress shielding [7]. This effect prevents proper activation of mechanosensitive ion channels and integrin‐mediated signaling cascades that are crucial for maintaining phenotype, leading to long‐term implant failure [8]. In addition, overly stiff scaffolds may induce mechanical trauma to surrounding tissue, promoting fibrosis and triggering adverse immune responses [9].

Given this, there is a dire need to process softer and more elastic materials that can better recapitulate soft tissue biomechanics [2, 10, 11, 12, 13]. Elastomers such as Poly(ethyleneoxideterephthalate)‐poly(butyleneterephthalate) (PEOT/PBT) (E ≈ 20–130 MPa) [2], Poly(L‐lactic acid)‐block‐Poly(ε‐caprolactone)‐block‐Poly(L‐lactic acid) (PLLA‐b‐PCL‐b‐PLLA) (E ≈ 1–2 MPa) [13], poly(urea‐siloxane) (E ≈ 27 MPa) [14], Poly(L‐lactide‐co‐ε‐caprolactone) (PLCL) (E ≈ 5–8 MPa) [12], and thermoplastic polycarbonate‐silicon polyurethane (TPCSUs) (E ≈ 1–3 MPa) [15] have recently been processed. In most cases, however, mechanical compliance is achieved through architectural design rather than intrinsic material behavior. This approach is suboptimal, as compliance gained through architectural changes inherently affects cell behavior, complicating the decoupling of mechanical and biological effects. Processing an inherently softer material, where compliance arises from bulk polymer behavior rather than scaffold architecture, therefore, represents a more desirable approach. This remains challenging, as MEW demands a narrow set of material prerequisites for reliable printing (see our earlier review) [16], where polymers beyond PCL exhibit markedly reduced achievable printing resolution [17, 18, 19, 20] and frequently undergo rapid thermal degradation at elevated temperatures, limiting scalability and clinical adoption [20].

To expand the material toolbox for MEW, we explore the MEW of polyethylene vinyl acetate (PEVA), a thermoplastic elastomer known for its flexibility, chemical stability, and biocompatibility [21]. PEVA is a semicrystalline copolymer composed of ethylene and vinyl acetate monomers, the latter of which imparts rubber‐like elasticity and modulates crystallinity [22]. PEVA is extensively utilized as a matrix material for controlled drug delivery [23], owing to its tunable release profiles governed by the vinyl acetate (VA) content. Reducing the VA fraction increases the amorphous character and decreases crystallinity, thereby enhancing matrix permeability and accelerating drug diffusion rates [23]. A prominent application of PEVA‐based drug delivery is in intravaginal rings (IVRs) for sustained release of hormones [24]. Among the limited number of polymers employed in commercial contraceptive IVRs, such as the NuvaRing, PEVA is distinguished by its favorable properties, including excellent biocompatibility, chemical inertness, minimal inflammatory response, and processability [25].

Despite PEVA's widespread use in biomedical applications, its integration into advanced biofabrication techniques, such as electrospinning, has proven challenging [26], and its potential in MEW remains entirely unexplored. Introducing PEVA into the MEW domain holds the potential to fabricate scaffolds with significantly greater compliance and elasticity than conventional polyester‐based structures. This is particularly relevant for engineering soft tissues, where mechanical mismatch between an implanted scaffold and the host tissue can impair integration and function [27]. Furthermore, PEVA's amenability to drug incorporation opens possibilities for designing MEW scaffolds with therapeutic functionality. By demonstrating the feasibility of PEVA processing via MEW, this work aims to broaden the material palette available for microscale scaffold fabrication and enable novel approaches to engineering mechanically adaptive biofunctional constructs.

In this study, we report the successful processing of PEVA via MEW using a custom‐built MEWron platform [28, 29], specifically engineered to accommodate the rheological and thermal characteristics of elastomeric polymers. It was necessary to optimize many processing parameters, including temperature, pressure, and electric field strength, to ensure stable jet formation and reproducible fiber deposition. We demonstrated high geometric precision in manufacturing microscale architectures by systematically analyzing fiber diameter, interfiber spacing, and vertical stacking. Furthermore, axial tensile and compression testing paired with preliminary biological assessments of cell attachment, biocompatibility, and alignment were conducted to determine the scaffold's potential in tissue engineering. Collectively, this work establishes a new processing window for PEVA in MEW and expands the range of materials available for the fabrication of mechanically compliant, spatially ordered scaffolds.

## 2 Results and Discussion

### 2.1 Material Analysis

**FIGURE 1:**
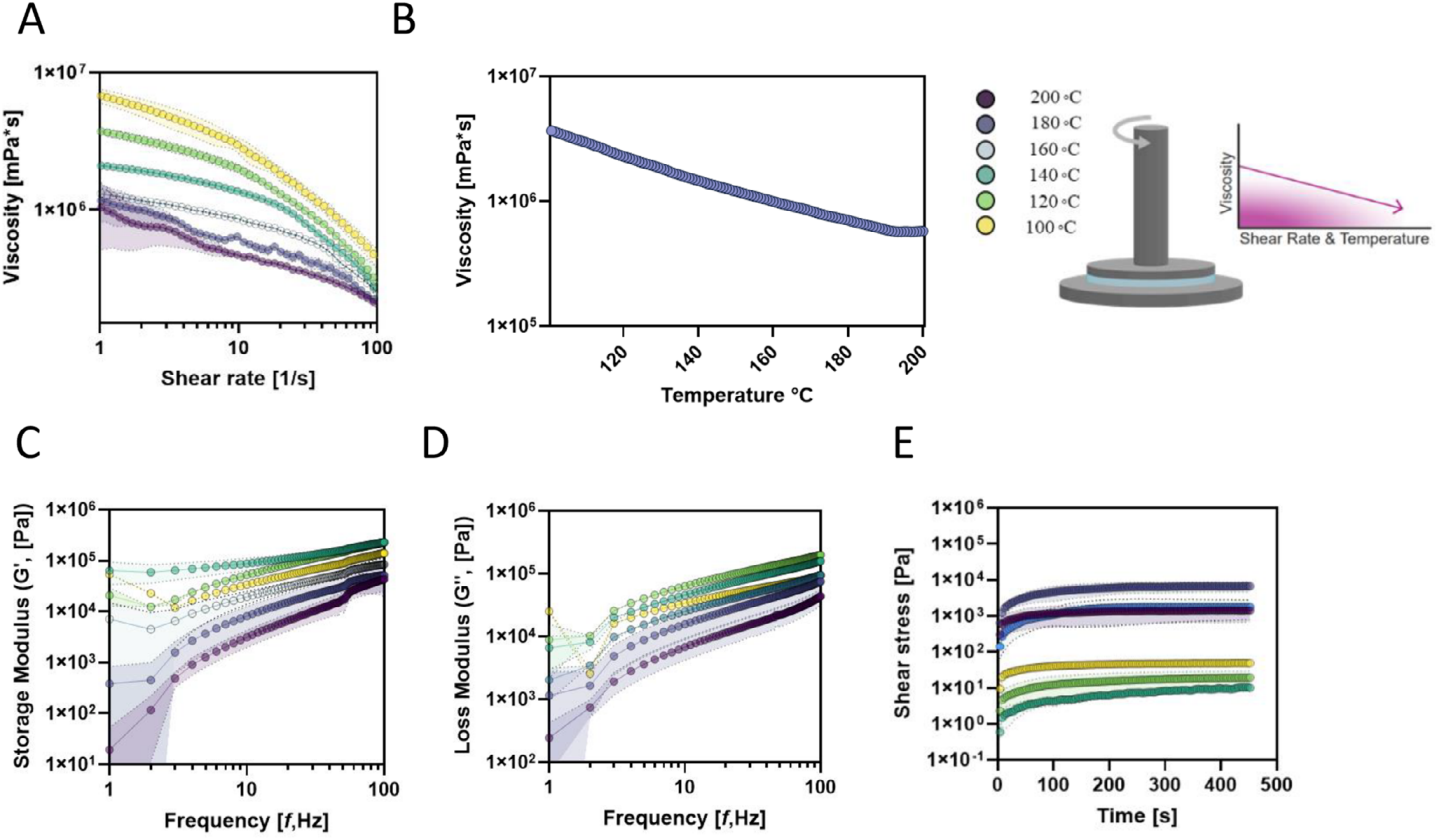
Rheological properties of PEVA were measured across a temperature range from 200°C to 100°C in 20°C increments.

PEVA was subjected to various temperatures (100°C–200°C) for analysis of viscosity. Viscosity measurements determined that viscosity declined over the shear rate range (1–100 [1/s]), with lower‐temperature datasets (yellow) having higher initial values and slower decay than higher temperatures (purple) (Figure 1A). This shear‐thinning behavior (depicted by the downward slope) is typical of pseudoplastic behavior in polymers. At higher shear rates, the viscosity presents a tendency toward a lower plateau, indicating complete chain alignment [30]. With temperature increment (yellow to purple, i.e., 100°C–200°C), the viscosity drop indicated reduced entanglement density correlated to an increased molecular motion; this drop in viscosity as a function of temperature was also observed under a constant shear (Figure 1B), suggesting a strong thermal sensitivity likely related to the amorphous PEVA phase softening and crystalline phase disruption [31]. By comparison, at the common dispensing temperature of 100°C [32], PCL exhibits a viscosity of approximately 1670 Pa·s, corresponding to the viscosity of PEVA at 134°C.

Storage (G′) and loss (G″) modulus as a function of frequency depicts that G’ and G″ were higher for low temperatures at low frequencies (Figure 1C,D). Higher frequencies (>10 Hz) revealed all temperatures to have a similar trend (the distance between them is narrower) and an upward slope, indicating a more elastic response (Figure S1) [33]. Increasing the temperature softened the material. This behavior was more pronounced at lower frequencies (where chain relaxation was more dominant) while at higher frequencies, the imposed deformation caused faster molecular rearrangements, diminishing the influence of temperature on G″. The increased variability observed at low frequencies (including the decrease in G′ and G″ around 2 Hz for 100°C samples) is attributable to the longer oscillation periods, which increase sensitivity to thermal drift, torque noise, and slow sample relaxation. In contrast, higher frequencies provide improved signal‐to‐noise ratios and more effective cycle averaging. To fully study the effect on G″ it was necessary to monitor the material behavior under a single shear rate over time (Figure S2), expressed in Figure 1E as shear stress. Under constant deformation, the shear stress test confirmed the results by showing higher stress retention at low temperatures and faster flow at high temperatures.

**FIGURE 2:**
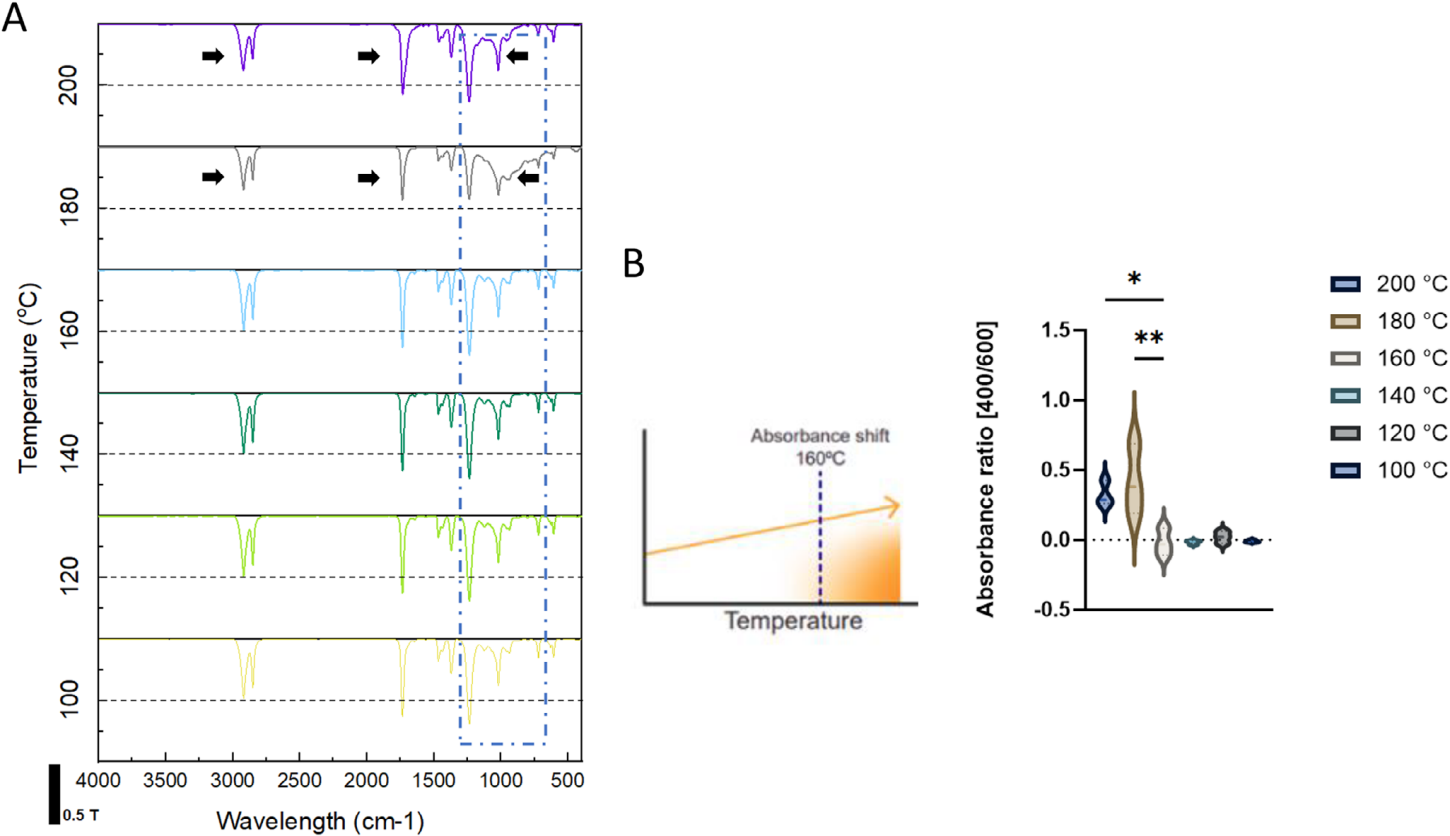
(A) FTIR transmittance spectra of PEVA following thermal degradation at various temperatures (scale bar 0.5 T), and (B) Absorbance ratio [400/600 nm] of thermally degraded PEVA.

FTIR analysis performed over the selected range of temperatures revealed that 100°C–200°C presents the characteristic peaks of PEVA deriving from the polyethylene (PE) segment and the VA components (Figure 2A). This included peaks at ∼ 2 916 and 2848 cm^−1^ corresponding to CH_2_ asymmetric and symmetric stretching, along with a peak at ∼1740 cm^−1^ assigned to C═O stretching of the acetate group [34]. The PEVA used contains 40% of VA, reflecting a balanced FTIR spectrum between the broadness and sharpness of PE and VA characteristic peaks. As hinted by the rheological behavior, higher temperatures showed a transition of material at higher temperatures, from 160°C to 200°C, the FTIR revealed a peak reduction of the CH_2_ bond (∼2916 cm^−1^), C═O (∼1740 cm^−1^), C─O stretching around 1100 cm^−1^ and >1000 cm^−1^ C─C, C─H. These changes suggest overall a chain scission and thermal degradation/oxidation [35, 36]. At 140°C, FTIR analysis revealed PEVA was thermally stable over 1 week of continuous heating, as indicated by no significant variations observed at PEVA characteristic peaks (Figure S3). Consistent with these findings, thermogravimetric analysis (TGA) showed significantly lower mass loss (< 0.25%) at 140°C compared to higher temperatures (160°C: 1.34%, 180°C: 1.8%, 200°C: 2.8%), indicating limited thermal degradation under these conditions (Figure S4).

When PEVA was exposed to high temperatures, it exhibited a visible shift in color toward yellow wavelengths, reflecting the process of oxidation. To quantify these changes, we performed absorbance spectroscopy measurements (Figure 2B) and normalized against transparent samples (100°C samples) as a reference. The quantification showed that the shift onset started from 160°C with high significance differences for 180°C and 200°C compared to 100°C; this result aligns with the observations from the FTIR and rheology analysis, as observed by other researchers [36].

### 2.2 Parameter Optimization

**FIGURE 3:**
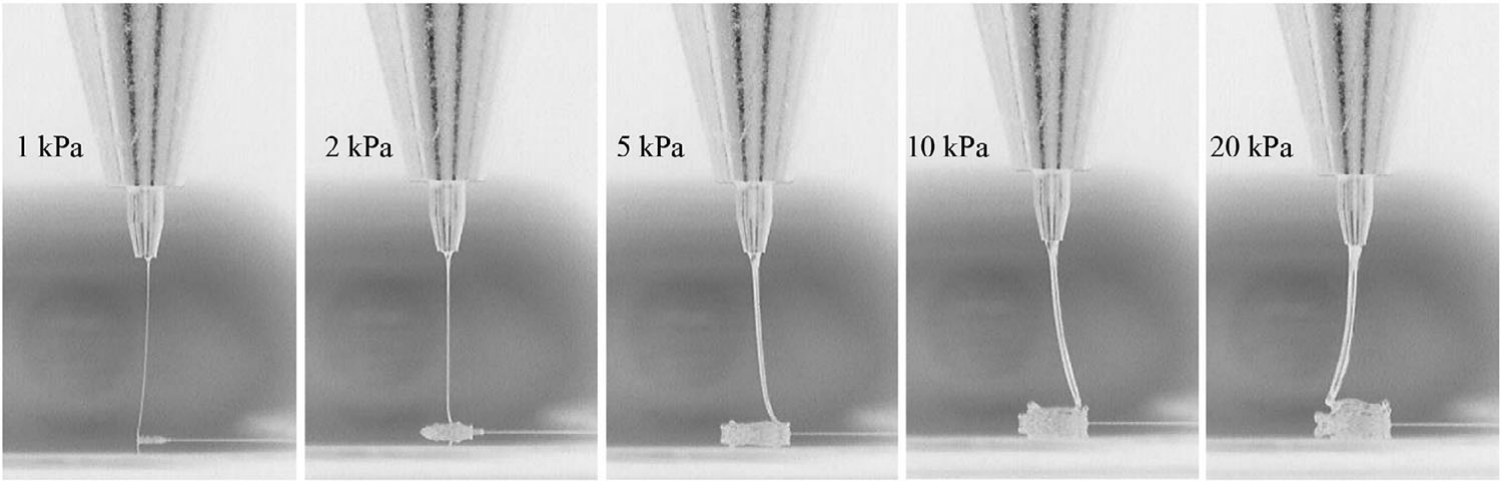
Effect of dispensing pressure on jet morphology (3 kV, 3 mm z‐height, stationary collector plate, 140°C).

PEVA demonstrated excellent processability across a broad temperature range. Stable jet formation was achieved at extrusion temperatures between 140°C and 200°C, where 100°C–120°C displayed rapid jet quenching and an inability to print. Under applied pressures ranging from 1 to 20 kPa (Figure 3), the material exhibited predictable flow characteristics, with jet stability maintained throughout the tested parameter space. During xy‐plane translation, the PEVA jet exhibited consistent lag behavior that varied systematically with translation speed (Movie S1).

Extended printing sessions at elevated temperatures led to progressive jet instability, with the viable printing window decreasing substantially above 180°C. FTIR and absorbance measurements elucidate that oxidation can be clearly identified as a dependency of temperature and is accompanied by visible yellowing of the polymer. The effects of thermal degradation were initially seen by a distinct increase in jet lag, irrespective of translation speed.

**FIGURE 4:**
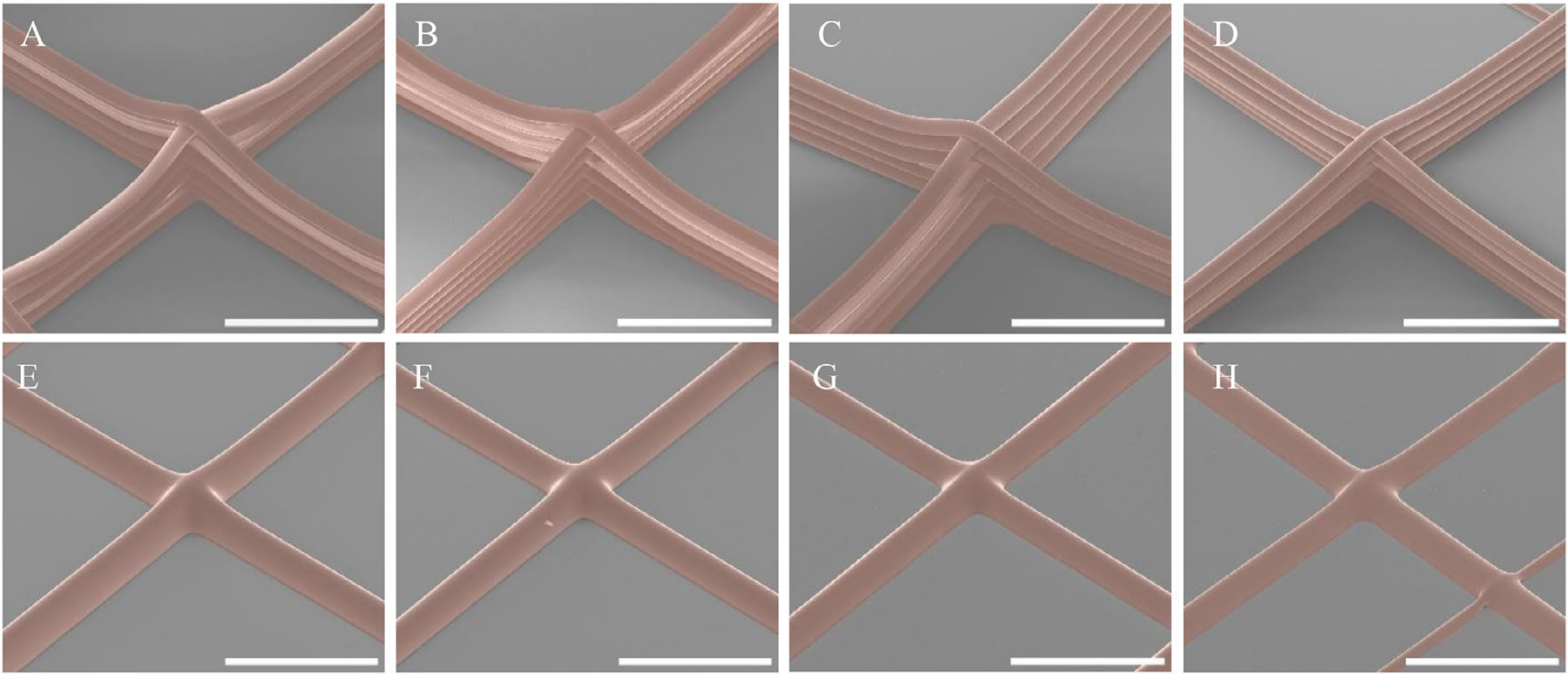
Effect of processing temperature on fiber integrity and bonding. (A–D) Fiber morphology at 140°C, 160°C, 180°C, and 200°C with an unheated collector plate (25°C). (E–H) Corresponding conditions with a heated collector plate (55°C) (scale bar: 500 µm).

At all tested dispensing temperatures, individual discrete fibers were successfully produced, confirming the effectiveness of the MEW process for PEVA (Figure 4A–D). Higher deposition temperatures yielded increased fiber diameters and a higher critical translation speed (CTS), advantageous for efficient fabrication of scaffolds (Table 1). Despite maintaining 1.1×CTS, jet lag increased up to 180°C and then decreased at 200°C, potentially due to the onset of thermal degradation (Table 1).

**TABLE 1:** Effect of dispensing temperature on CTS (2.5 mm z‐height, 2 kPa, 3 kV), with corresponding jet lag and fiber diameter measured at 1.1 × CTS.

T (°C)	CTS (mm/min)	Jet lag (°)	Ø_fiber_ (µm)
140	25	9	18
160	52	12	30
180	95	18	30
200	110	14	34

Heating the collector plate to 55°C caused fiber coalescence as the PEVA remained in a molten state upon deposition, resulting in merged layering that compromised the discrete fiber deposition essential for scaffold production (Figure 4E–H). In contrast, unheated collectors (25°C) promoted rapid solidification, preserving individual fiber morphology. Based on these findings, 140°C was selected as the optimal dispensing temperature, as it represents the lowest temperature at which stable fiber formation could be achieved while providing a melt viscosity most comparable to the gold‐standard PCL. Selecting the lower temperature, however, results in a very slow CTS, approximately 0.3 mm/s (Table 2), due to relatively high viscosity and jet quenching, reducing the jet's responsiveness to the electric field. For more rapid printing, the issue of material degradation may be bypassed by incorporating a gear extrusion system instead of the pneumatic pressure‐based extrusion used here [37]. As gear‐driven extrusion confines thermal exposure to the localized melt zone rather than a bulk polymer reservoir, it substantially mitigates thermal degradation while enabling highly controlled, low‐volume flow rates. If opting for a gear extrusion system, however, a gear ratio reduction is required, as MEW requires lower extrusion rates compared to standard 3D printing [38].

**TABLE 2:** Effect of z‐height and dispensing pressure on CTS at a constant electric field strength (1.2kV/mm), with corresponding jet lag and fiber diameter measured at 1.1xCTS.

Z‐height	P (kPa)	CTS (mm/min)	Jet lag (°)	Ø_f_ (µm)
1.5	1	9	18	27
	2	5	11	40
	5	3	11	63
	10	2	17	120
	20	1	11	208
2.5	1	29	26	16
	2	25	19	18
	5	22	22	29
	10	15	16	50
	20	8	17	81
3.5	1	29	25	11
	2	26	18	15
	5	24	22	25
	10	18	15	40
	20	11	7	67

At a dispensing temperature of 140°C, the influence of z‐height and dispensing pressure on the CTS was systematically investigated (Table 2). To ensure consistent electrostatic loading, the electric field strength was fixed by maintaining a 1.2 kV/mm z‐height. For all z‐heights, reduced pressures resulted in increased CTS. This behavior is expected because lower pressures produce finer jets that experience greater electrostatic acceleration, necessitating higher translation speeds to maintain linear fiber deposition. To analyze jet lag and fiber morphology, fibers were printed at 1.1×CTS derived from the CTS measured for each condition. In all cases, fiber diameters decreased at lower pressures due to reduced flow rates, with a further reduction at higher z‐heights, attributable to increased electrostatic stretching over longer nozzle–collector distances.

Although z‐heights below 1.5 mm are common in literature [39], PEVA exhibited poor jet stability under these conditions. Droplet accumulation at the nozzle and intermittent pulsing indicated insufficient tensile force to initiate stable jet formation despite maintaining a constant electric field strength. Lowering the dispensing pressure reduced but could not eliminate this instability, highlighting a material‐dependent limitation on minimum workable z‐height. Despite the clear and independent influences of pressure and z‐height on CTS and fiber diameter, jet lag showed no strong parametric dependence. Instead, it remained internally consistent within each condition set, suggesting that PEVA's viscoelastic response governs jet lag more strongly than the geometric or electrical parameters explored here.

Accordingly, a z‐height of 2.5 mm was determined as optimal to avoid the unstable jet observed at 1.5 mm, and the risk of premature fiber solidification and increased reactivity to electrostatic distortion at 3.5 mm. These parameters provided sustained printing capability with minimal observed variations in CTS or fiber diameter over one week (Table S1), consistent with TGA and FTIR analyses indicating negligible PEVA thermal degradation during continuous heating and printing at 140°C.

### 2.3 Achievable Resolution

Following the tuning of printing temperatures, subsequent experiments were conducted to determine the range of viable fiber diameters and interfiber spacing – two factors known to affect key biological outcomes [40]. It has been reported that minimizing fiber diameter and interfiber spacing typically requires low z‐heights and high voltages, with high translation speeds instrumental in thinning the jet [41]. Accordingly, and in reference to the optimized printing parameters, the z‐height was set to 2.5 mm, while the applied voltage was maintained at 3 kV, where further increases resulted in immediate arcing.

**FIGURE 5:**
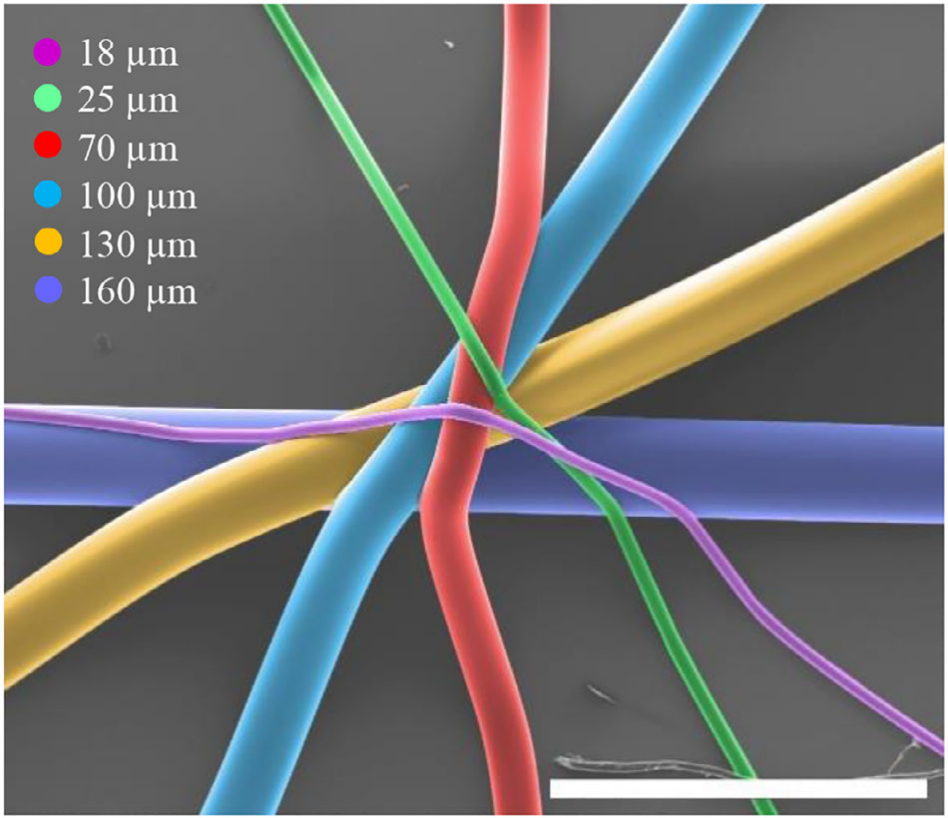
Fiber diameter dependence on dispensing pressure (scale bar: 550 µm).

Fiber diameter variations were visualized in a radial star pattern. This setup was used to assess the effect of pressure on fiber morphology. Pressures were gradually reduced from 20 to 1 kPa while maintaining a translation speed of 1.1×CTS. Corresponding fiber diameters decreased from approximately 160–18 µm, measured at pressures of 20, 15, 10, 5, 2, and 1 kPa, respectively (Figure 5). Increasing the translation speed to 5xCTS further reduced fiber diameter (≈ 8 µm); however, this resulted in excessive jet lag that restricted the accurate deposition of more complex non‐linear architectures.

This range of fiber diameters spans a physiologically relevant scale when compared to various natural tissue structures. For instance, the finest fibers produced at 1 kPa (∼18 µm) are comparable to the size of capillaries (7–9 µm) [42], Harversian canals (∼50 µm) [43], venules (8–100 µm) [44], arterioles (5–100 µm) [45], and muscle fibers (100 µm) [46] among others, suggesting potential for structural biomimicry across a wide range of native tissues. The ability to tune fiber diameter through pressure modulation provides a means of tailoring scaffold architecture to match the dimensions of specific tissue components, critical for promoting appropriate cellular interactions and tissue integration.

One primary limitation associated with EHD fabrication techniques is fiber bridging. Fiber bridging occurs when deposited fibers distort the uniform electric field enough to attract dispensing material, merging two fibers that were intended to be deposited as independent fibers. The distorted electric field is dependent on the thickness and geometry of the fiber already on the collector plate, and thus, achieving a low inter‐fiber spacing is increasingly difficult with thicker fibers. To this end, the highest reported diameter to interfiber spacing ratio (0.29) was achieved by Zhang et al., who fabricated 60 µm pores using 10–20 µm diameter PLA fibers [47]. To the best of our knowledge, this is the only reported sub‐100 µm resolution achieved for a material other than PCL.

**FIGURE 6:**
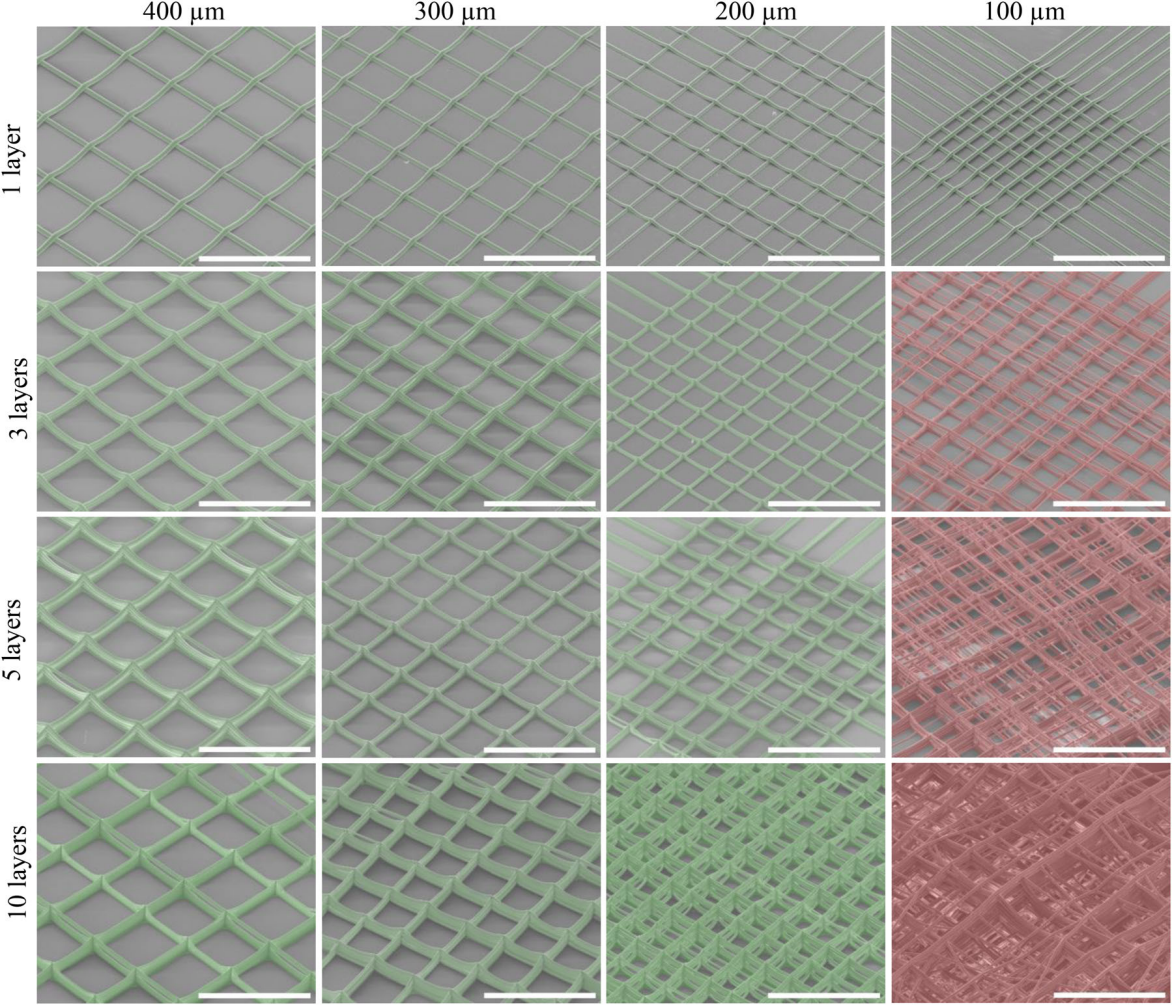
Interfiber spacing and layering potential of MEW PEVA (green: correctly deposited fibers, red: misplaced fibers). All scaffolds printed with 2.5mm z‐height, 3 kV, 140°C, 1.1xCTS, 1 kPa (scale bar: 500 µm).

**FIGURE 7:**
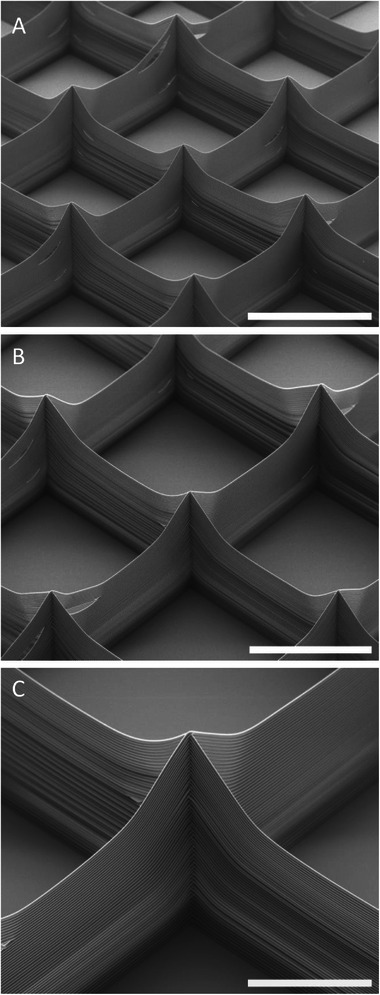
Depiction of highly precise layering of a 50‐layer box‐shaped PEVA scaffold. Scale bar: (A) 2 mm, (B) 1 mm, and (C) 500 µm.

Scaffolds consisting of 400, 300, 200, and 100 µm interfiber spacing were fabricated with 1, 3, 5, and 10 layers (Figure 6). Further layering was not investigated due to the extended print time (> 48 h). Fiber bridging was absent in all single‐layer scaffolds, first appearing at layer 3 in 100 µm pores, while 200, 300, and 400 µm pores remained free of bridging through 10 layers. Comparatively, the printing resolution demonstrated here surpasses that of other elastic materials, including PEOT/PBT (500 µm) [2], PLLA‐b‐PCL‐b‐PLLA (500 µm) [13], PLCL (600 µm) [12], TPU (300 µm) [10], TPCSUs (200 µm) [15], poly(urea‐siloxane) (1 mm) [14], and provides one of the highest diameter to interfiber spacing ratio to date (0.23 ± 0.06), comparable to the current highest reported ratio of 0.29 [47]. Despite minimal interfiber attraction, there were increasing instances of fiber repulsion with decreased interfiber spacing. The variability in spacing was quantified using the standard deviation of interfiber distances, yielding 27, 33, 26, and 14 µm for the 100, 200, 300, and 400 µm spaced pores, respectively. These findings highlight the capability of PEVA to produce high‐resolution, multilayered scaffolds with structurally consistent pore networks across a broad range of interfiber spacings. To demonstrate the scalability of MEW PEVA, a thicker 50‐layer scaffold (1.5 mm thick) was fabricated, exhibiting highly precise and repeatable fiber stacking (Figure 7).

### 2.4 Mechanical Characterization

Monotonic tensile testing is one of the most commonly employed methods for characterizing the mechanical behavior of materials, particularly in soft tissue and biomaterials research. It provides direct insight into how a material responds to uniaxial stress under controlled strain, enabling quantification of key parameters such as stiffness (E), yield strain (ε_y_), and ultimate tensile strength (UTS). To highlight comparisons of newly processed PEVA, PCL fibers (printed at 100°C, 3 mm z‐height, 3 kV, 3 mm s^−1^) were used as a gold standard control due to their well‐established processibility, while MEW TPU (printed at 240°C, 3mm z‐height, 3 kV, 1 mm s^−1^) fibers served as an elastic control to benchmark soft, stretchable behavior, with increasing prevalence in MEW processibility [10, 11, 28, 48].

The tensile properties of all three materials varied drastically (Table 3), with PEVA exhibiting the most compliance (E: 11.40 ± 0.98 kPa), approximately 4‐fold more than TPU (E: 47.45 ± 3.56 kPa) and 35‐fold more than PCL (E: 377.25 ± 74.92 kPa). Statistically (Figure S5), there was no difference between the yield strain of PEVA (44.05 ± 1.43%) and TPU (42.28 ± 2.64%), both outperforming PCL (13.85 ± 0.72%). This result is expected, as PCL is known for its stiff mechanical behavior due to its semi‐crystalline structure and limited chain mobility [49], whereas PEVA and TPU contain more flexible, amorphous segments that allow greater molecular movement and extensibility under load [50, 51]. PEVA did however exhibit a significantly lower work til yield (Wy) (122.60 ± 16.80 kJ/m^3^) compared to both TPU (495.60 ± 113.00 kJ/m^3^) and PCL (385.40 ± 76.51 kJ/m^3^), indicating reduced energy absorption before plastic deformation.

**TABLE 3:** Summary of mechanical properties obtained from monotonic tensile loading of MEW fibers at 0.1 mm s^−1^ (*n* = 3).

	E (kPa)	ε_y_ (%)	W_Y_ (kJ/m^3^)	UTS (MPa)	SAF (%)
PEVA	11.40 ± 0.98	44.05 ± 1.43	122.60 ± 16.80	> 1.30	> 400
TPU	47.45 ± 3.56	42.28 ± 2.64	495.60 ± 113.00	> 4.90	> 400
PCL	377.25 ± 74.92	13.85 ± 0.72	385.40 ± 76.51	7.85 ± 1.05	> 400

**FIGURE 8:**
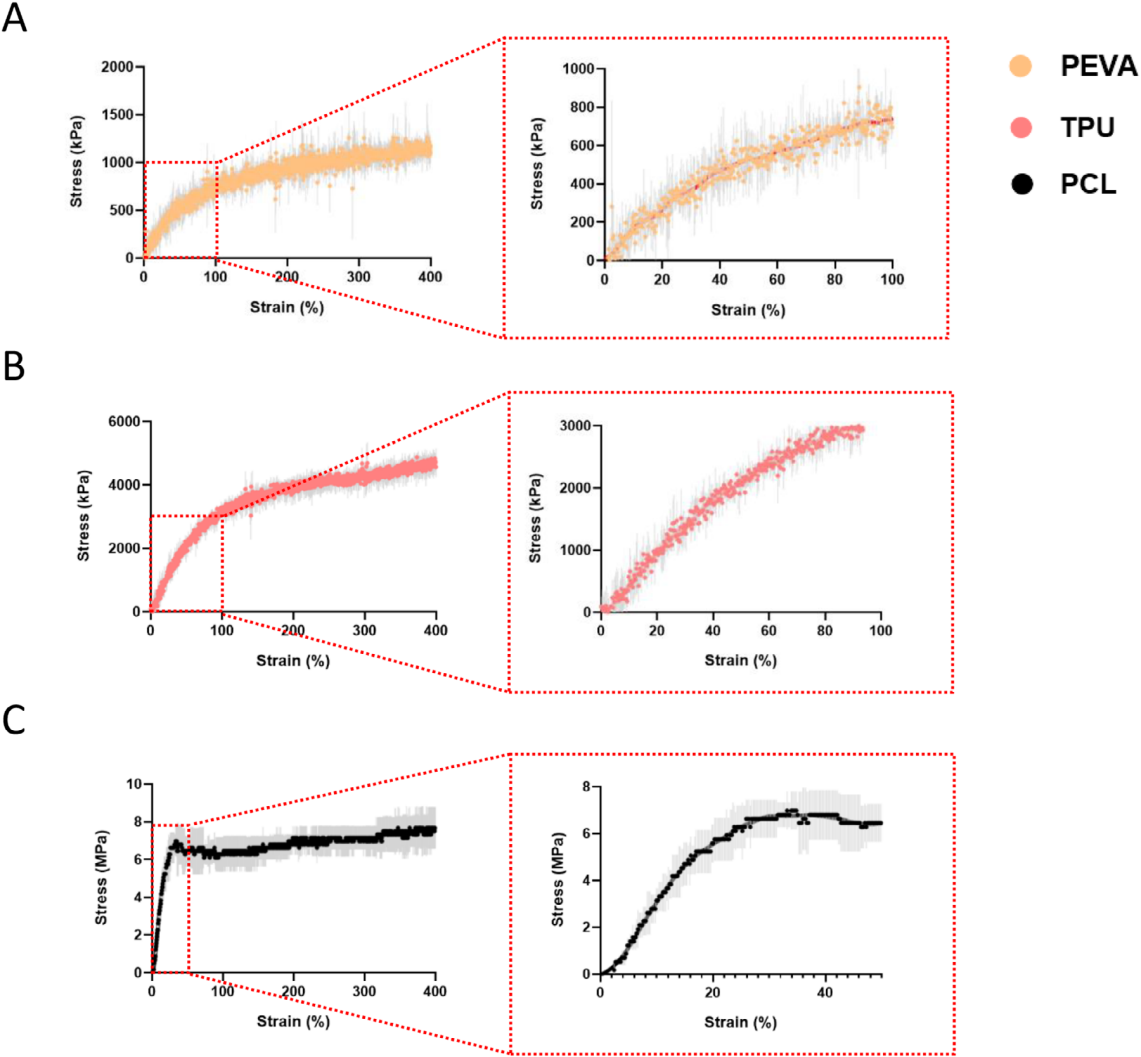
Stress–strain curves of MEW (A) PEVA, (B) TPU, and (C) PCL fibers exposed to monotonic tensile loading at 0.1 mm s^−1^ (*n* = 3).

Given the maximum extension of the TA Electroforce 5500 mechanical loading device's shaft was 11 mm, both PEVA and TPU did not exhibit rupture or even a plateau in stress within the applied 400% strain, and thus strain at failure (SAF) (PEVA and TPU: > 400%) and UTS (PEVA: > 1.3 MPa, TPU: > 4.9 MPa) were unable to be determined decisively (Figure 8). PCL exhibited a UTS of 7.85 ± 1.05 MPa, with SAF > 400%, where necking initiated at the center of the samples and propagated outward, consistent with typical ductile polymer behavior [52]. Alternative scaffold architectures were unable to obtain distinct force readings and thus were not analyzed under tensile conditions.

For tissues that predominantly experience compressive loading under physiological conditions, tensile testing alone is insufficient, as polymers often exhibit asymmetric mechanical responses under tension and compression. Accordingly, monotonic compression testing was conducted using 20‐layer box‐shaped scaffolds (1 mm pore size), a representative MEW architecture commonly employed in literature [53]. Owing to the substantially longer print durations required for these multi‐layer scaffolds compared to the single‐fiber constructs used for tensile analysis, TPU underwent rapid thermal degradation (within 1 h) and could not be fabricated reproducibly. As such, compressive properties were evaluated by comparing PEVA scaffolds directly to PCL, serving as a stable and process‐reliable control.

**FIGURE 9:**
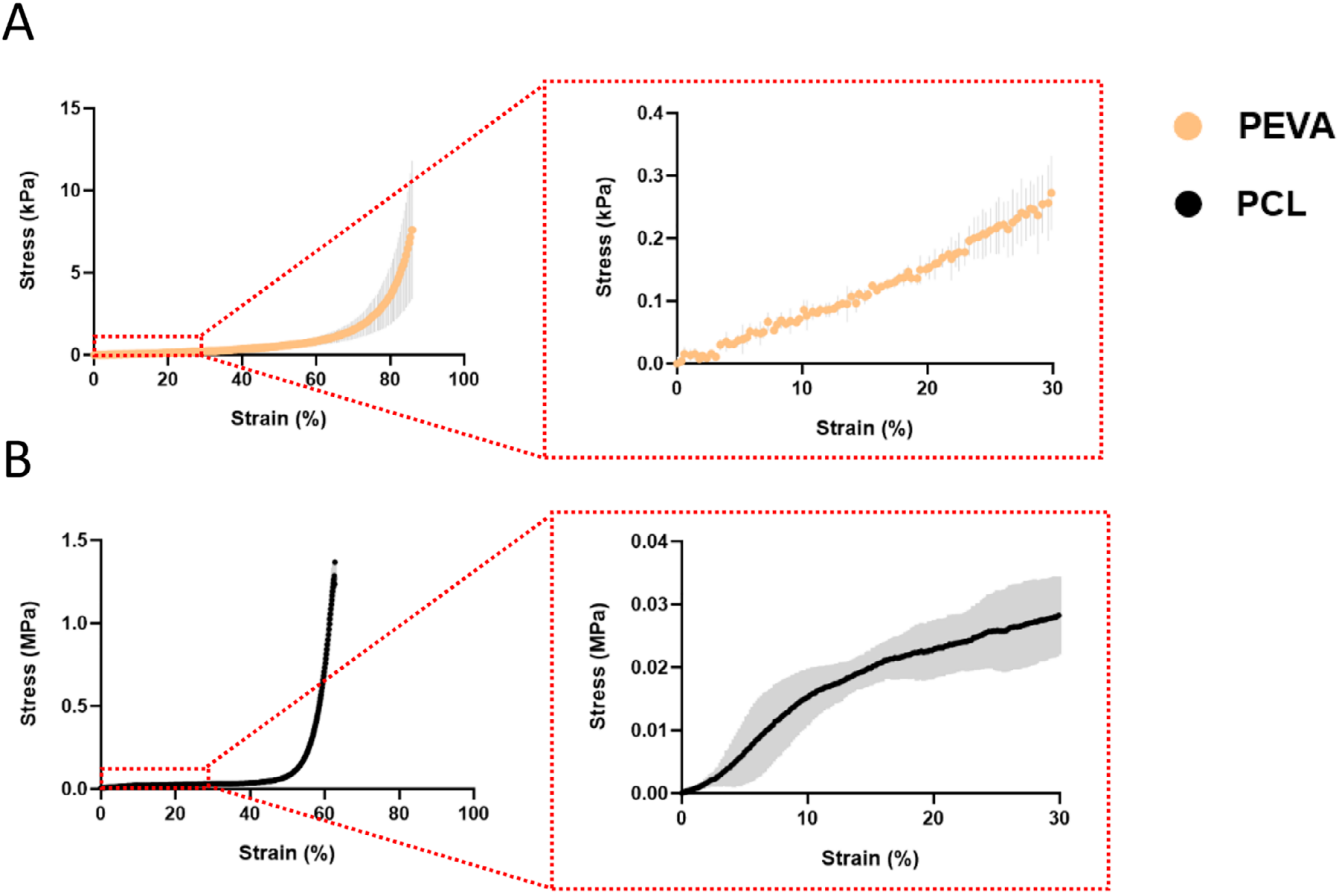
Stress–strain curves of MEW (A) PEVA and (B) PCL scaffolds exposed to monotonic compression loading at 0.1 mm s^−1^ (*n* = 3).

Both PEVA and PCL displayed an extended toe region, exceeding ∼80% and ∼55% strain, respectively (Figure 9). This behavior is attributed to the scaffold architecture, in which overlapping filament intersections create localized regions of increased height. During the initial phase of compression, contact occurs first at these elevated nodes, which deform downward before load is transferred to the lower‐lying filaments. As these intersection points collapse, they impose axial loading on the adjacent vertical wall‐like fibers, resulting in a prolonged low‐stiffness region prior to full structural engagement. Taken from the linear region prior to the onset of the toe region, PEVA exhibited an exceptionally low compressive modulus (E: 1.07 ± 0.07 kPa), which increased to 105.70 ± 12.76 kPa after full structural engagement (Table 4). No yielding was observed within the applied strain window (ε_y_: > 85%), consistent with the highly compliant, large‐strain behavior characteristic of PEVA under compression [54]. Although PCL had a similar toe region, there was a distinct yield point (ε_y_: 8.60 ± 1.05%), in which modulus obtained prior to yielding (E: 282.20 ± 71.82 kPa) was statistically (Figure S6) greater than PEVA. As this yielding occurred prior to the toe region, it is assumed that yielding occurred through deformation of the elevated nodes at fiber intersections, indicating microscopic yielding as opposed to macroscopic yielding typical at the onset of full structural engagement. Neither PEVA nor PCL reached catastrophic failure within the maximum compressive strain limits of the instrument; however, PEVA supported stresses of 9.79 ± 3.20 kPa at 85% strain, while PCL sustained stresses of 1.37 ± 0.01 MPa at 65% strain.

**TABLE 4:** Summary of mechanical properties obtained from monotonic compression loading of MEW scaffolds at 0.1 mm s^−1^ (*n* = 3).

	E (kPa)	ε_y_ (%)	W_Y_ (kJ/m^3^)	UTS (units defined below)	SAF (%)
PEVA	1.07 ± 0.07 – 105.70 ± 12.76	> 85	> 0.99 ± 0.20	> 9.79 ± 3.20 kPa	> 85
PCL	282.20 ± 71.82	8.60 ± 1.05	0.48 ± 0.15	> 1.37 ± 0.01 MPa	> 65

MEW PEVA's tensile properties align with the stiffness of many soft tissues, such as skeletal (1.75–18 kPa) [55] and smooth muscle (5–50 kPa) [56, 57], the hypodermis (15 kPa) [58], myocardium (8–15 kPa) [59], liver (12.16 ± 1.20 kPa) [60], and the median range for all lung tumors (12.73 kPa) [61]. Similarly, MEW PEVAs compressive properties align with the stiffness of even softer tissues such as various brain tissues (1.19–9.46 kPa) [62], bone marrow (0.25–24.7 kPa) [63], and adipose tissue (1.60–2.90 kPa) [64]. As cells consistently respond more favorably to substrates that mimic the stiffness of their native environment [65], PEVA provides a mechanically compatible platform for soft tissue engineering. This is particularly advantageous for mechanosensitive cells, where subtle variations in substrate stiffness can modulate fate decisions through mechanotransduction pathways [66, 67].

Furthermore, with compelling evidence that physical forces play a critical role in tissue development during embryogenesis [68], the shift toward dynamic culture systems is gaining traction. To replicate one specific aspect of the biophysical cues experienced during development, dimension‐based mechanical loading, such as uniaxial or biaxial strain, is often incorporated into bioreactor designs [69]. To date, MEW scaffolds have seen limited application in dynamic cultivation environments and, to the best of our knowledge, have only been exposed to shear stress (via perfusion bioreactors) in models of the trabecula and tunica intima [70, 71], magnet‐induced bending for skeletal muscle [72], and uniaxial strain for the controlled stimulation of tenocytes [73]. In such mechanically active systems, PEVA's unique mechanical properties present distinct advantages over conventional MEW materials. The combination of low stiffness and high extensibility positions PEVA scaffolds as optimal substrates for mechanically active culture environments, where the scaffold must maintain structural integrity while allowing cells to experience physiologically relevant mechanical stimuli without the constraint of overly rigid matrix materials that could impede natural tissue development. This phenomenon, known as stress shielding, reduces cellular mechanostimulation by diverting applied loads through overly stiff scaffold materials [74]. PEVA's low modulus may mitigate this effect, enabling effective force transmission to cells, supporting tissue maturation in dynamic culture environments.

Another potential application of MEW PEVA fibers is in the fabrication of flexible microelectrode arrays (MEAs), particularly those used as neuromuscular implants. Intramuscular implants are inherently vulnerable to mechanical stress, as they must endure repeated deformation and displacement associated with muscle contraction and length changes. Addressing the resulting mechanical mismatch typically involves tailoring the Young's modulus of interface materials. In an optimal design, the mechanical properties of the implant, particularly the Young's modulus, would closely approximate those of skeletal muscle to minimize interfacial strain. However, skeletal muscle is inherently soft, with a modulus in the kilopascal range, contrasting sharply with the gigapascal‐range stiffness of conventional electrode materials. While mechanical compliance is essential post‐implantation to accommodate continuous muscle deformation, a higher stiffness is typically required during insertion to enable precise placement and minimize buckling. Recently, Xue et al. utilized MEW TPU fibers sputter‐coated with 5 nm titanium and 250 nm platinum, creating a flexible MEA platform for myocardial tissue electrophysiological signal recording [75]. Given PEVA's significantly lower elastic modulus and higher print resolution compared to TPU, PEVA‐based MEAs offer a closer mechanical match to skeletal muscle, minimizing mismatch that can lead to implant failure and tissue damage. Enhanced print resolution also enables the fabrication of finely defined electrode geometries, improving signal targeting and neural integration. Moreover, PEVA's exceptional extensibility (strain at failure > 400%), intrinsic compliance, and non‐degradable nature make it well‐suited for accommodating the dynamic mechanical environment of contracting muscle while preserving electrical functionality.

### 2.5 Cell Culture

Transferring multilayer scaffolds from the build plate to the cell culture plate proved problematic. Ethanol was required to detach scaffolds from the collector plate; however, it frequently induced folding, resulting in self‐adhesion and loss of usability. This effect was minimized through lower ethanol concentrations; however, the efficacy as a release agent was diminished. An optimized process was developed, where samples were slid directly from the silicon wafer (via 70% ethanol) into a well plate (pre‐filled with ethanol). This process was reliable and allowed scaffolds to naturally sink to the bottom of the well plate. Importantly, despite the soft and compliant nature of PEVA, fibers did not become crimped once exposed to ethanol, as previously demonstrated in thinner PCL fibers [32]. Instead, multilayer fibers remained straight and preserved their pore architecture through several (PBS) washes and one week of cell culture. While these in vitro observations suggest that multilayer PEVA scaffolds can maintain architectural integrity under hydrated conditions at 37°C, definitive assessment of long‐term mechanical stability in vivo will require implantation studies, which are beyond the scope of the present work.

**FIGURE 10:**
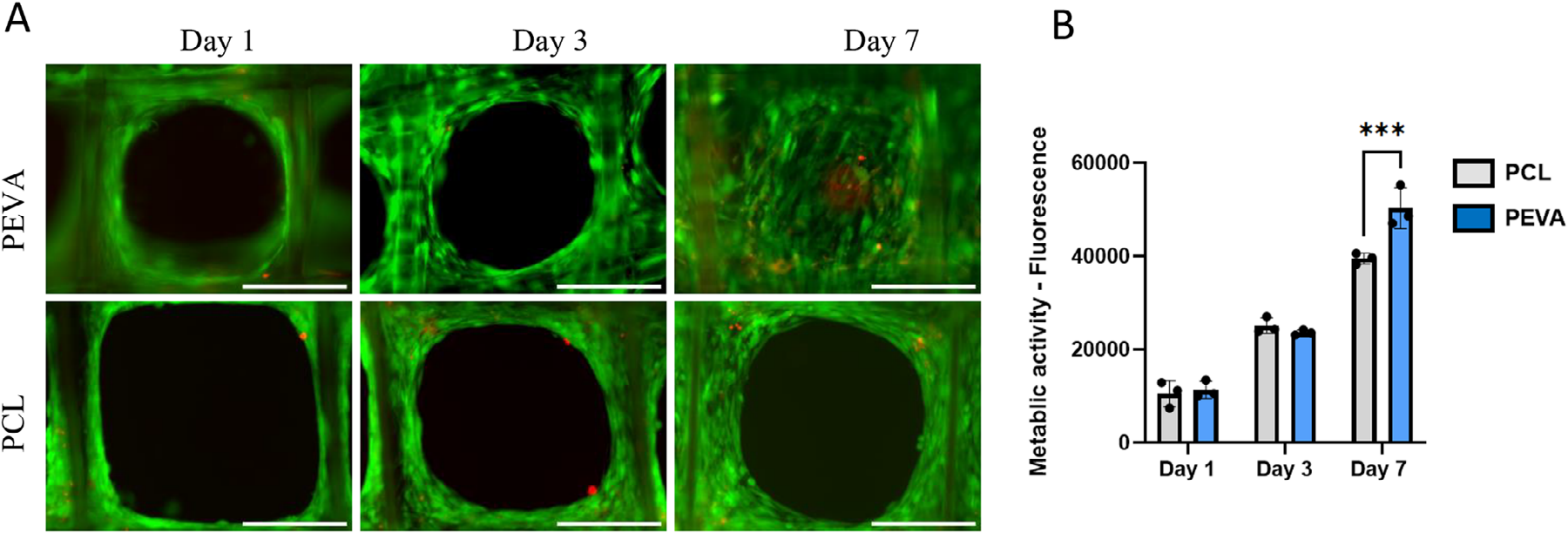
(A) Live (green) and dead (red) staining of primary human skeletal muscle myoblasts seeded onto PEVA and PCL scaffolds (5 layers, 0.5 mm interfiber spacing, box scaffold) (scale bar: 200 µm). (B) Corresponding metabolic activity of cells on PEVA and PCL scaffolds through 1 week of cell culture.

Live/Dead staining confirmed that MEW‐fabricated PEVA scaffolds supported robust adhesion and viability of primary human skeletal muscle myoblasts (Figure 10A). Cell attachment and proliferation did not differ significantly between PEVA and PCL scaffolds at days 1 and 3. By day 7, PEVA scaffolds demonstrated a statistically significant increase in metabolic activity compared with PCL (Figure 10B), suggesting enhanced cell proliferation. This delayed divergence in metabolic activity may reflect the markedly lower stiffness of PEVA compared with PCL, as compliant substrates can allow greater cell‐generated deformation and altered mechanotransductive signaling over time, even in the absence of externally applied mechanical stimulation [76, 77]. Cellular adhesion was consistently concentrated at fiber junctions, an observation in agreement with prior studies, where enhanced topographical cues and increased interconnectivity of pores facilitate initial cell anchorage [78]. Over time, cells proliferated and expanded radially to occupy adjacent pore spaces, forming concentric cellular patterns characteristic of isotropic filling. This radial migration and pore colonization behavior mirrors the tissue growth dynamics predicted by the computational growth model established by VandenHeuvel et al., underscoring the influence of scaffold architecture on guiding spatial cellular organization [79].

**FIGURE 11:**
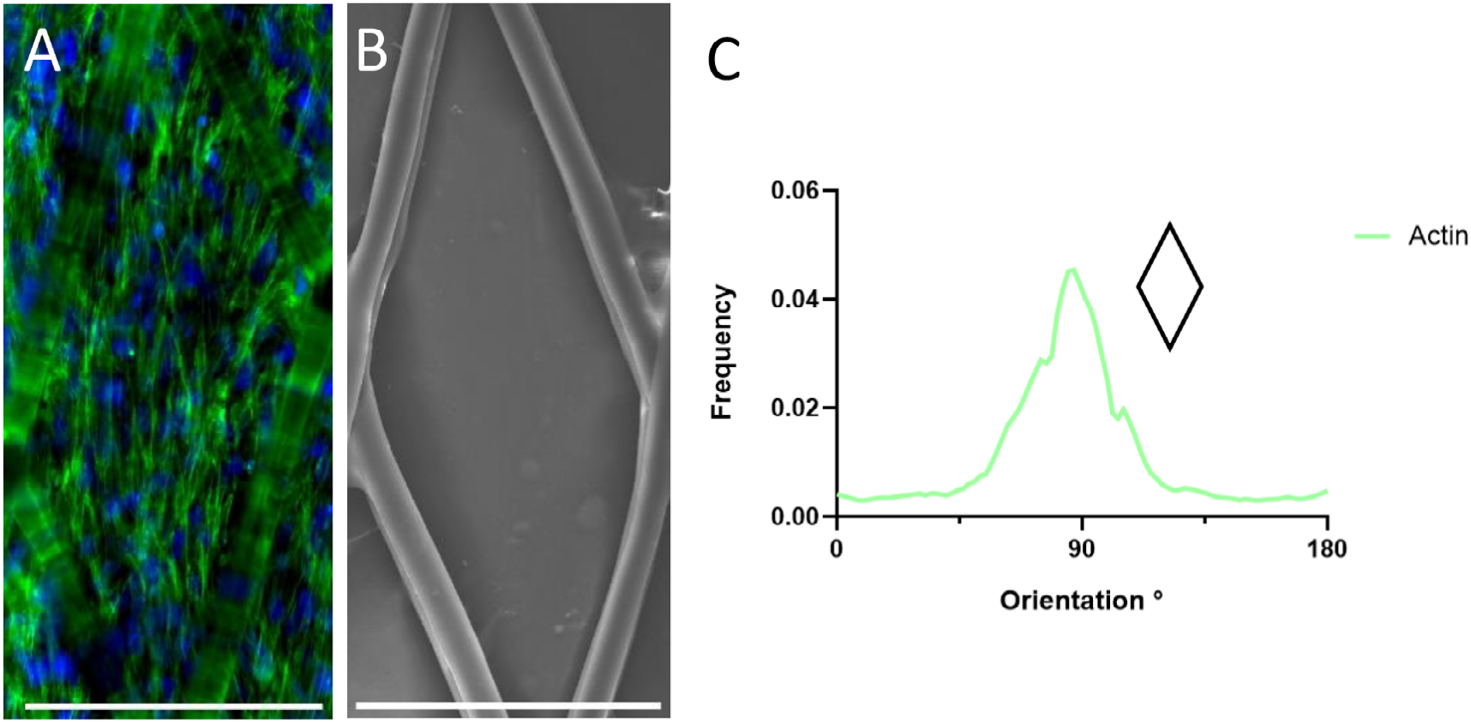
(A) Aligned cell growth induced by 20° PEVA scaffolds (green: phalloidin, blue: DAPI), and (B) corresponding SEM image depicting a cell‐filled pore (scale bar: 200 µm). (C) Directionality histogram of actin filaments in a 20° PEVA scaffold, smoothed using a second‐order polynomial fit (*n* = 3).

PEVA scaffolds fabricated with a 20° fiber orientation promoted pronounced alignment of myoblasts along the longitudinal axis of the resulting diamond‐shaped pores (Figure 11A). Building upon previous findings demonstrating the capacity of 20° offset architectures to guide cellular organization [32], the PEVA scaffolds supported robust alignment of myoblast actin filaments (21.97° ± 3.82°) as quantified via the full‐width half maximum (FWHM) method (Figure 11B). The ability of PEVA scaffolds with a 20° fiber orientation to induce highly aligned myoblast cytoskeletal organization is of particular relevance to musculoskeletal tissue engineering, where native tissues such as skeletal muscle, tendon, and myocardium display pronounced anisotropy at both the cellular and extracellular matrix levels [80]. Recapitulating this structural alignment in vitro is critical for guiding cell fate, promoting lineage‐specific maturation, and ultimately restoring functional tissue architecture. Aligned myoblasts facilitate the formation of elongated, multinucleated myotubes with enhanced contractile properties, while anisotropic ECM deposition supports mechanical coherence and directional signal transduction [81]. The capacity of these scaffolds to direct actin filament organization, therefore, represents a key biomimetic feature, offering a platform conducive to functional soft tissue development and integration in vivo.

Collectively, the low elastic modulus and high yield strain of PEVA make it well suited for soft tissue applications where minimizing stress shielding and accommodating repeated elastic deformation are critical. The observed cell proliferation and predictable growth behavior further support the integration of PEVA into existing MEW design strategies. Moreover, given PEVA's extensive use in drug delivery applications, drug‐loaded PEVA MEW scaffolds represent a logical and promising direction for future development. While non‐degradable PEVA is suitable for certain tissue engineering applications, the development of degradable formulations would substantially broaden its applicability. Although efforts have been made to develop biodegradable variants of PEVA, these studies predominantly focused on environmental degradation, such as breakdown in soil or compost conditions [82]. Such analyses, while relevant for ecological considerations, fail to recapitulate the complex enzymatic, hydrolytic, and cellular processes governing biodegradation within physiological environments. This leaves a significant gap in understanding PEVA's potential bioresorption in vivo; thus, we propose that efforts be made to engineer biodegradable PEVA, potentially via copolymer synthesis, to accelerate its adoption in tissue engineering [83]. Despite its non‐biodegradable nature, PEVA has proven cytocompatible, and, after 3 months in PBS solution at 37°C, surface degradation was evident, and supported by significant reductions in tensile properties [84]. Furthermore, considering in vivo applications, PEVA demonstrated long‐term (6‐month) biocompatibility in the pelvic cavity of various species (rats, rabbits, and rhesus monkeys), with no apparent foreign body reaction [85]. Collectively, PEVA demonstrates substantial promise as a MEW scaffold material for guided soft tissue regeneration, and could further benefit from future strategies to introduce controlled biodegradability under physiologically relevant conditions.

## 3 Conclusion

In this study, we report the first MEW of PEVA, advancing the repertoire of biomaterials available for high‐resolution scaffold fabrication. Through detailed rheological characterization and systematic variation of processing parameters, we identified optimal MEW processing parameters for PEVA (140°C, 2.5 mm z‐height, 3 kV, 1 kPa, 1.1xCTS), with viable print time exceeding 1 week. These conditions yielded fiber diameters down to 8 µm, with bridging observed only from layer 3 in 100 µm pores, while larger 200, 300, and 400 µm pores remained structurally stable through 10 layers. A diameter‐to‐interfiber spacing ratio of 0.23 ± 0.06 was produced, signifying the highest reported ratio for elastic MEW polymers. PEVA fibers exhibited the most tensile compliance (E: 11.40 ± 0.98 kPa), approximately 4‐fold more than TPU (E: 47.45 ± 3.56 kPa) and 35‐fold more than PCL (E: 377.25 ± 74.92 kPa). Statistically, there was no difference between the tensile yield strain of PEVA (44.05 ± 1.43%) and TPU (42.28 ± 2.64%), both outperforming PCL (13.85 ± 0.72%). Under compression, macroporous PEVA scaffolds demonstrated an elongated toe region up to 75%, with roughly 250‐fold greater compliance than PCL, and a yield strain exceeding 85%. Biologically, PEVA scaffolds supported robust cell attachment and survival, with initial growth dynamics analogous to PCL and a significant increase in metabolic activity by day 7. Moreover, established scaffold designs successfully guided the alignment of primary human skeletal muscle myoblasts' actin filaments (21.97 ± 3.82°) as quantified by the FWHM method. Collectively, this work introduces PEVA as a novel and promising polymer for MEW, broadens the material options for precision MEW, and sets the stage for tailored scaffold architectures in tissue engineering.

## 4 Experimental Section

### 4.1 Materials

PEVA containing 40 wt.% VA, with a melt flow index of 57 g/10 min (measured at 190°C under a 2.167 kg load), was purchased from Sigma–Aldrich (St. Louis, MO, USA). The polymer contained 200–800 ppm butylated hydroxytoluene (BHT) as an antioxidant stabilizer. PURASORB PC 12 (Corbion, The Netherlands) was used as the control, serving as the gold standard for MEW printing [78], while Pellethane 2363–80A TPU elastomer (Lubrizol LifeSciences) served as an elastic control. Prior to processing, all materials were dehumidified to eliminate moisture‐induced variability in thermal behavior and jet stability. PEVA and TPU pellets were dried at 50°C for 5 h in a vacuum oven (≤5 mbar), followed by a controlled ramp‐up in temperature at a rate of 10°C/min until the target temperature was reached. The molten PEVA and TPU were then held at this temperature for 1 h to ensure thermal equilibration before initiating deposition. Similarly, PCL was dehumidified at 40°C for 5 h under vacuum, then heated at the same rate (10°C/min) to its respective temperature. Unlike PEVA, PCL was held isothermally for 24 h prior to MEW processing to achieve consistent filament deposition and print fidelity as previously described [32].

### 4.2 Rheology

An Physica MCR 301 Rheometer (Anton Paar, Germany) was used to determine the viscosity of PEVA in two modes, first the rotational shear‐viscosity was performed with a shear rate ranging from 0.01 to 1000 s^−1^ for 100°C–200°C in 20°C increments, next to compare the temperature dependency under constant shear, the viscosity was monitored at a shear rate of 10 s^−1^ using a temperature ramp from 100°C to 200°C. Time‐sweep measurements at low deformation regime (1% strain and 1 rad s^−1^ frequency) were applied to investigate the change in elastic (G’) and viscous modulus (G’) over time at temperatures varying from 100°C to 200°C using incremental steps of 20°C. Frequency sweep measurements were in a ramp mode from 1 to 100 Hz, recording the storage and loss modulus as a function of the frequency change for all temperatures ranging from 100°C to 200°C in 20°C increments. The average of triplicates and standard deviations was plotted using Prism 10.1 software (GraphPad Inc., US).

### 4.3 Fourier Transform Infrared Spectroscopy (FTIR)

Samples subjected to various temperatures ranging from 100°C to 200°C for 4 h, or heated at 140°C for 1 week to assess degradation over time, were evaluated using FTIR by transmittance measurement using the average of 64 scan (wavelength from 400 to 4000 cm^−1^, resolution of 4 cm^−1^) with an ATR‐FTIR Bruker instrument (Bruker Co., US). Stacked transmittance curves were plotted using OriginLab software (OriginLab Co., US).

### 4.4 Thermogravimetric Analysis (TGA)

Thermogravimetric analysis was performed using a TGA 209 Libra (Netzsch). Approximately 5 mg of each sample was placed in a crucible and heated from 30 to the target temperature (140°C, 160°C, 180°C, or 200°C) at a rate of 10°C min^−1^ under an air atmosphere, followed by an isothermal hold for 4 h. Mass changes were recorded as a function of temperature. To emulate prolonged thermal exposure during MEW, samples preheated at 140°C for 1 week were analyzed up to 300°C and compared with unheated controls.

### 4.5 Oxidation Monitoring using Absorbance Measurement

PEVA was placed on top of a glass coverslip (0.16 mm thickness) and melted using a heating plate at temperatures from 100°C to 200°C in 20°C increments for 4h. After this time, the samples were allowed to reach room temperature, then the samples (*n* = 3) were retrieved and placed on a 12‐well plate for absorbance spectrum measurements performed using a matrix scan (10 × 10 mm) using a CLARIOStar Plate Reader (GMG Labtech GmbH, Germany). The absorbance ratio (400/600 nm) and difference against 100°C (transparent sample) were plotted using GraphPad Prism 10.1 software Inc (US).

### 4.6 MEW Set‐Up

A custom MEWron system, as described in depth by Reizabal et al., was utilized for all printing (MEWron, Supporting Information) [28]. Briefly, compressed air was regulated using an AD 3000C unit (Iwashita Instruments) rated up to 100 kPa. All printing was conducted with a 200 µm internal diameter nozzle, at a 2.5 mm z‐height, with a high voltage print head and grounded collector plate (Gamma High Voltage Research). The original voron0.2 heated print bed was maintained (rated up to 120°C), and the magnetic stainless steel base plate was replaced with a silicon wafer due to its conductive, flat, and scratch‐resistant properties. A physical emergency stop button was added to the front‐left corner of the printer frame, housed in a custom 3D‐printed red PLA enclosure for visibility. The button operates dual rails: one in‐line with the HV supply's AC input and the other connected to the controller's digital input. When activated, the E‐stop interrupts power to the HV supply and triggers a software‐controlled shutdown sequence, which disables all motion, heating, and high‐voltage components until the system is manually reset. Additionally, magnetic reed switches were installed and programmed to disable translational motion once a door opens, providing another layer of operator protection.

### 4.7 Printing Parameters Optimization

To define optimal processing conditions for PEVA, a tiered parameter selection strategy was employed on a simple box scaffold. An experimental matrix comprising eight regimes was designed to balance coverage and efficiency. Four dispensing temperatures (140°C, 160°C, 180°C, and 200°C) were combined with two collector temperatures (23°C and 50°C) to explore the thermal boundaries for stable fiber formation and stacking. The nozzle‐to‐collector distance was fixed at 2.5 mm, with an applied voltage of 3 kV. Translation speed was set to 1.1× the CTS – the minimum speed required to achieve continuous, non‐buckling fiber deposition, where the pressure was varied to maintain comparable fiber diameter. Following selection of dispensing and collector temperatures, the nozzle‐to‐collector distance (z‐height) was optimized by assessing fibre diameter, jet lag, and CTS at z‐heights of 1.5, 2.5, and 3.5 mm.

### 4.8 Achievable Resolutions

Following initial parameter screening, the regime exhibiting the most stable and uniform fiber deposition was selected to investigate the resolution limits of MEW PEVA. To probe spatial resolution, pneumatic pressure, applied voltage, and translation speed were systematically varied to minimize fiber diameter while maintaining process stability. Fiber diameter was evaluated using a radial star pattern, with fibers deposited at 30° intervals converging at a central point [86].

Single‐layer, box‐shaped scaffolds with varying interfiber spacings (400, 300, 200, and 100 µm) were used to determine the smallest repeatable pore size achievable without fiber coalescence or distortion (G‐codes, Supporting Information). To guide this evaluation, we adopted the method described by Du et al., who established reliable parameters for high‐resolution MEW scaffolds [87]. Specifically, turning loops were implemented with a diameter 10 times the inter‐fiber spacing between parallel fibers, with a turning speed of 0.625×CTS. Additionally, the [gcode_arcs] command was added to the mainsails configuration file to enable interpretation of G2 and G3 commands.

Multilayer scaffolds incorporating all previously described pore sizes were fabricated by stacking 1, 3, 5, and 10 layers. Layering was continued until the onset of visible printing defects, such as fiber misalignment, loss of geometric fidelity, or deformation due to accumulated thermal or mechanical stress. This approach enabled the identification of the maximum achievable stacking height at high spatial resolution, thereby defining the operational limits of MEW PEVA.

### 4.9 Mechanical Characterization

Mechanical characterization was performed at room temperature using a TA Electroforce 5500 mechanical loading device (TA Instruments, New Castle, DE, USA) fitted with a 2.5 N load cell for PEVA and TPU, and a 225 N load cell (Honeywell, Columbus) for PCL. Tensile properties were assessed using single fibers of assumed circular cross‐section with a gauge length of 2.5 mm, whereas compressive properties were evaluated using 20‐layer, box‐shaped scaffolds with 1 mm pore spacing. All tests were conducted under monotonic loading at a strain rate of 0.1 mm s^−1^ and standardized to the effective cross‐sectional area of the scaffold measured prior to testing, enabling stress‐strain data to be calculated. Results were analyzed to define Young's modulus (E), yield strain (ε_y_) via the 0.2% strain offset method, work till yield (W_Y_), ultimate tensile strength (UTS), and strain at failure (SAF).

### 4.10 Cell Culture

This work was performed in accordance with Australian National Statement on Ethical Conduct in Human Research guidelines (SVHM HREC Ref: 096/01, SVHM HREC Ref: 093/21). Primary human skeletal muscle myoblasts were expanded in Hams/F10 (TRACE) supplemented with 20% fetal bovine serum (Invitrogen), 2.5 ng mL^−1^ bFGF (PeproTech Asia), 2 mm L‐glutamine (Invitrogen), 100 U mL^−1^ of penicillin (Invitrogen), and 100 mg mL^−1^ streptomycin (Invitrogen).

Once T‐75 flasks were 70%–80% confluent, cells were trypsinized and centrifuged at x 800 g for 5 min to pellet the cells and finally re‐suspended at a concentration of (840 × 10^3^ cells/mL) in 600 µL media. 100 µL (84 × 10^3^ cells) of the cell suspension was then seeded into each well of a 24‐well ultra‐low attachment cell culture plate, featuring a 3D printed PLA anchor, reducing the seeded area to 32 mm^2^, equivalent to that of a 96‐well plate.

### 4.11 Live/Dead Assay

After days 1, 3, and 7, live/dead staining was conducted on 5‐layer box scaffolds with 0.5 mm pores to ensure biocompatibility and cell survival of myoblasts on MEW scaffolds. Identification of live and dead cells was carried out via calcein AM (ThermoFisher Scientific Inc.) and ethidium homodimer (ThermoFisher Scientific Inc.) staining, as per the manufacturer's instructions. All specimens were visualized using an Olympus IX70 fluorescent microscope and images obtained using Spot Advanced 4.0.9 software (Diagnostic Instruments) and processed in ImageJ.

### 4.12 Cellular Metabolic Activity

To quantify the attachment and proliferation of cells on these scaffolds, CellTiter‐Blue (CTB) Reagent (Promega, Madison, WI, USA) was used according to the manufacturer's instructions. On days 1, 3, and 7, spent media was removed, and scaffolds were washed twice in fresh media to remove any unattached cells. Media was then replaced with a solution containing a 1:4 ratio of CTB:media and incubated for 2 h at 37°C and 5% CO_2_. Solution was collected in an OptiPlate 96F and measured in a CLARIOStar plate reader at 550−15 nm excitation and 600−20 nm emission. A control without cells was included to correct for the background fluorescence of the media.

### 4.13 Staining of Actin Filaments

For labeling of actin filaments, the media was removed, and cells were fixed in 4% paraformaldehyde (Sigma) for 60 min, followed by staining with AlexaFluor 488 Phalloidin (Invitrogen) according to the manufacturer's instructions. Nuclei were counterstained with 1 µg mL^−1^ DAPI (4,6‐diamidino‐2‐phenylindole dihydrochloride, Sigma) for 5 min, and specimens were mounted in fluorescent mounting medium (Dako) for microscopy.

### 4.14 Alignment Analysis

To induce alignment, cells were cultured on 3‐layer scaffolds with a 20° fiber offset, as previously described [32]. The alignment of myoblast actin filaments was quantified using the FWHM approach. The FWHM represents the width of the peak at half of its maximum height in the alignment histograms generated by the ImageJ Directionality plugin and is defined in Equation (1) [88].

(1) $$FWHM\left(X\right)=2\sqrt{2ln2\sigma }$$

Here, σ denotes the standard deviation of the Gaussian distribution, describing the spread of the data or curve, where $2\sqrt{2ln2}$ is a constant factor arising from the process of finding where the Gaussian curve reaches half of its maximum value. A narrower FWHM corresponds to stronger alignment, as it indicates that most cells are oriented within a small angular range. For this analysis, the FWHM was calculated automatically by fitting the histogram data with a Gaussian distribution, as implemented in OriginPro (2021b).

### 4.15 Scanning Electron Microscopy

The microscopic morphology of fabricated MEW scaffolds was analyzed by SEM. Biological constructs were fixed with 2.5% glutaraldehyde (high purity) in 0.05 m cacodylate buffer, pH 7.4, for 1 h. Subsequent washes in 0.1 m phosphate buffer (3 × 15 min) and distilled water (3 × 5 min) were concluded with an ETOH dehydration process before SEM imaging. All samples were sputter‐coated with iridium (5 nm) and imaged using Quanta SEM QFEG. SEM images were obtained using 20 kV accelerating voltage, under high vacuum, with a spot size of 5. Color was applied post‐acquisition using Adobe Photoshop (Adobe Inc., 2025) for visual enhancement.

### 4.16 Statistics

All statistical analysis was performed using GraphPad Prism version 9, with all data presented as mean ± standard deviation (S.D.). A parametric approach was chosen based on the continuous nature of the data, and one‐way analysis of variance (ANOVA) was applied with Tukey post hoc analysis to assess pairwise comparisons. The assumption of homogeneity of variances was determined, and statistical significance was defined as *p* < 0.05 (^*^ *p* < 0.05, ^**^ *p* < 0.01, ^***^ *p* < 0.001).

## Funding

The authors would like to acknowledge the National Health and Medical Research Council (Ideas Grants 2002723 and 2038179) and FSHD Global Research Foundation Grant Number 53, for supporting this work. F.S. is supported by an RMIT STEM Scholarship.

## Conflicts of Interest

The authors declare no conflicts of interest.

## Supporting information




**Supporting File 1**: adhm70949‐sup‐0001‐SuppMat.docx.

**Supporting File 2**: adhm70949‐sup‐0002‐MovieS1.wmv.

**Supporting File 3**: adhm70949‐sup‐0002‐Data.zip.

## Data Availability

The data that support the findings of this study are openly available at the following https://doi.org/10.5281/zenodo.18652202.
