# Supplementary material for: Melt Electrowriting High Resolution Poly(ethylene‐co‐vinyl acetate) Scaffolds for Soft Tissue Engineering
Source: Adv Healthc Mater. 2026 Feb 24;15(17):e04945. doi: 10.1002/adhm.202504945 (PMC13175299; doi:10.1002/adhm.202504945)
Supplement: Supplementary file 1 — Supporting File 1: adhm70949‐sup‐0001‐SuppMat.docx. [file ADHM-15-0-s001.docx]

Figure S1. PEVA Storage and Loss modulus frequency sweep at various temperatures.

Frequency-dependent analysis reveals that at low frequencies, G″ becomes increasingly prominent with rising temperature, particularly at 180–200 °C, indicating long-time relaxation processes and increased viscous dissipation under slow deformation. In contrast, at high frequencies, G′ remains dominant across all temperatures, reflecting the persistence of elastic responses associated with short-time chain dynamics and physical constraints. The absence of a G′-G″ crossover within the measured frequency range suggests that PEVA retains an elastic response even at 200 °C. These trends demonstrate that temperature primarily controls the balance between elastic storage and viscous dissipation, while frequency governs whether short- or long-timescale molecular motions dominate the mechanical response.


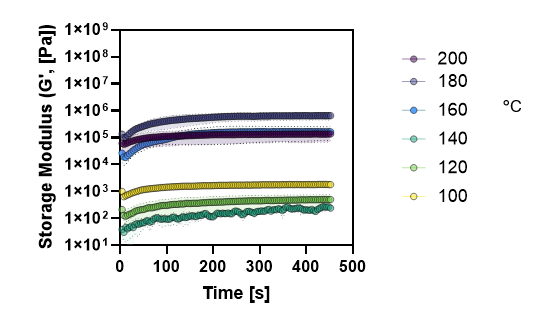

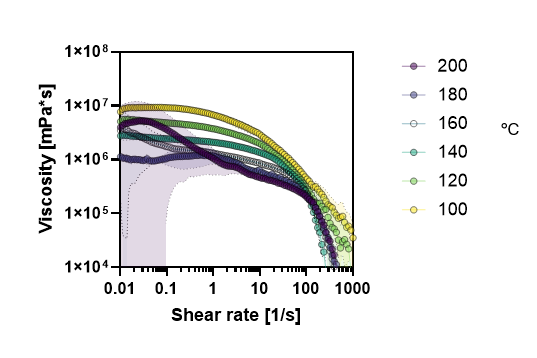

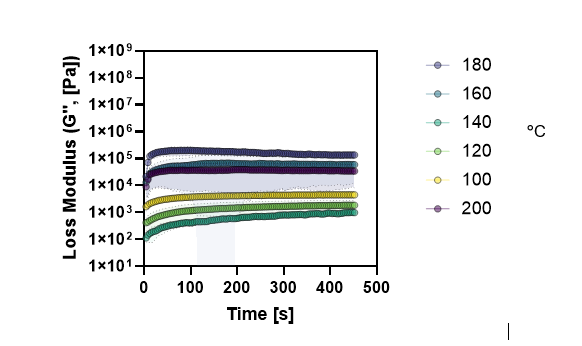


Figure S2. Loss modulus [Pa] and Storage modulus [Pa] monitored over time, and viscosity at starting at low shear rates (0.01 [1/s]) up to high shear rates (1000 [1/s]).

Across 140–200 °C temperature range, PEVA exhibits a systematic reduction in both storage (G′) and loss (G″) moduli with increasing temperature, consistent with progressive thermal softening. At 140–160 °C, G′ remains higher than G″ over the full frequency window, indicating predominantly elastic, solid-like behaviour governed by restricted chain mobility and residual crystalline ethylene domains. Increasing the temperature to 180 °C leads to a marked decrease in G′ and a relative increase in G″, narrowing the modulus gap. At 200 °C, both moduli are substantially reduced, and the dominance of G′ is further weakened, consistent with a transition toward a highly rubbery or melt-like viscoelastic regime.

Samples under 140 °C temperature regimes spanning from 24 - 7 days showed minor peak modifications compared to the control samples (not subjected to temperature), with no significant variations observed at PEVA characteristic peaks (i.e. ~1200, ~1250 and ~1000 cm-1) among the samples, suggesting that a chemistry modification at this temperature over the evaluated time, is unlikely.


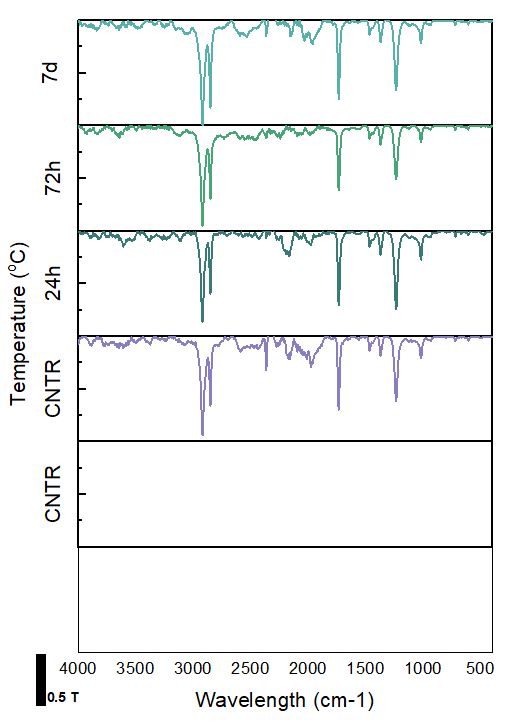

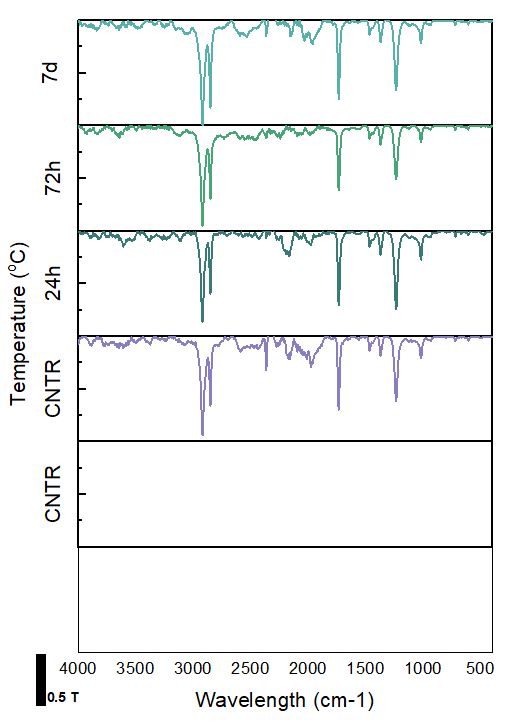


Figure S3. FTIR analysis conducted on PEVA samples heated at 140 °C for 1 week.


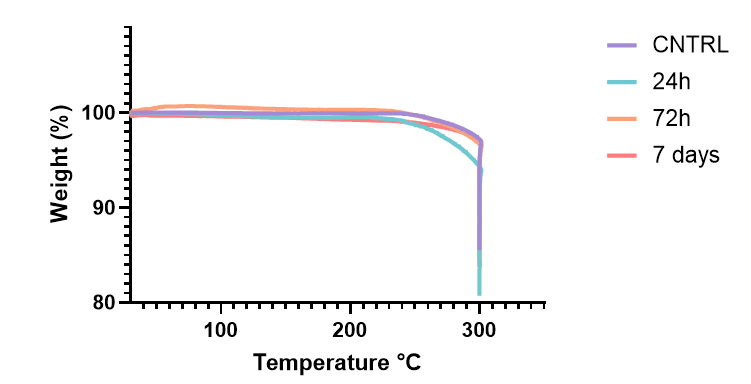

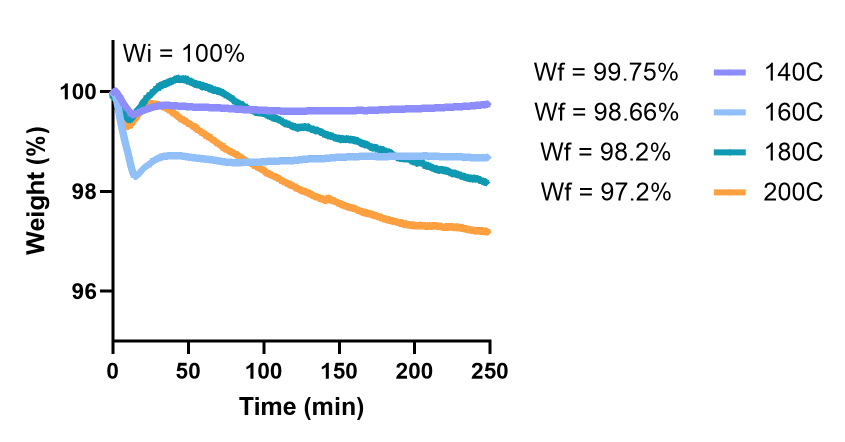


Isothermal thermogravimetric analysis of PEVA revealed a clear temperature-dependent mass loss over the 4 h hold period. The average mass-loss rate increased from −0.057 %·h⁻¹ at 140 °C to −0.672 %·h⁻¹ at 200 °C, indicating thermally activated degradation kinetics. At 140 °C, the minimal total mass loss (<0.25%) suggests that PEVA remains largely stable over extended exposure, with the observed change likely dominated by desorption of residual volatiles. In contrast, samples held at 160–200 °C exhibited sustained and progressively faster mass loss, consistent with the onset and acceleration of vinyl acetate deacetylation, accompanied by acetic acid evolution. The absence of a clear mass-loss plateau at higher temperatures indicates that this process proceeds continuously over hour-long timescales rather than occurring as a discrete event. Notably, exposure at 200 °C resulted in nearly 3% mass loss, implying chemical modification during prolonged thermal exposure. With up to 1-week continuous heating at 140 °C, samples did not significantly affect the polymer degradation rate under a wide thermal range (30-300 °C). This result indicates no significant modification of the polymer backbone when submitted to our MEW conditions.


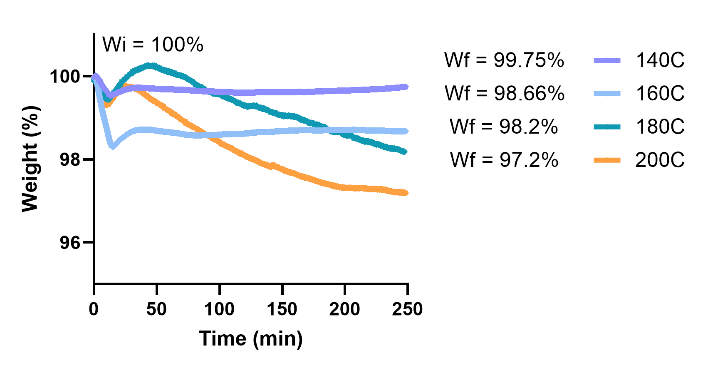


140 °C

160 °C

180 °C

200 °C

Figure S4. Isothermal thermogravimetric analysis of PEVA A) exposed to 4 hours of constant heat at various temperatures, and B) ramped temperature of PEVA pre-heated for 1 week at 140 °C.

Table S1. Stability of PEVA printing parameters through 1 week of continuous printing.

| Day | P (kPa) | CTS (mm/min) | Jet lag (°) | | Ø_f_ (μm) | |
| --- | --- | --- | --- | --- | --- | --- |
| 1 | 1 | 29 | | 26 | | 16 |
|  | 2 | 25 | | 19 | | 18 |
|  | 5 | 22 | | 22 | | 29 |
|  | 10 | 15 | | 16 | | 50 |
|  | 20 | 8 | | 17 | | 81 |
| 3 | 1 | 29 | | 22 | | 14 |
|  | 2 | 25 | | 17 | | 17 |
|  | 5 | 22 | | 17 | | 27 |
|  | 10 | 15 | | 13 | | 48 |
|  | 20 | 8 | | 7 | | 80 |
| 7 | 1 | 32 | | 15 | | 15 |
|  | 2 | 27 | | 8 | | 17 |
|  | 5 | 23 | | 14 | | 27 |
|  | 10 | 15 | | 9 | | 46 |
|  | 20 | 8 | | 13 | | 78 |


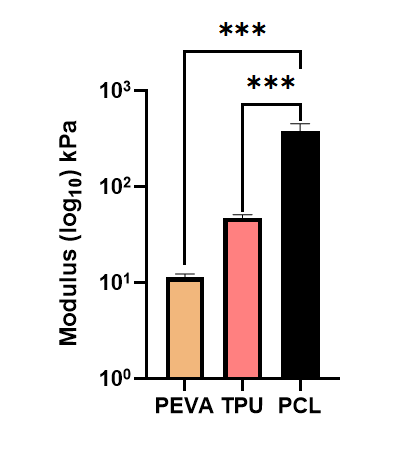

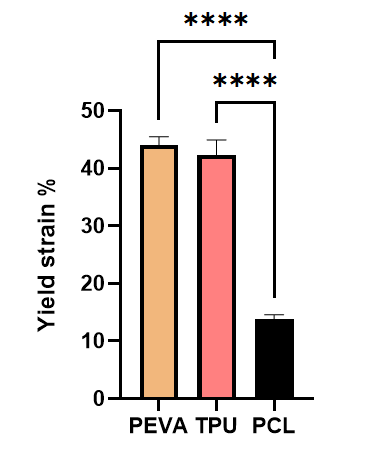


Figure S5. Statistical analysis of tensile properties.


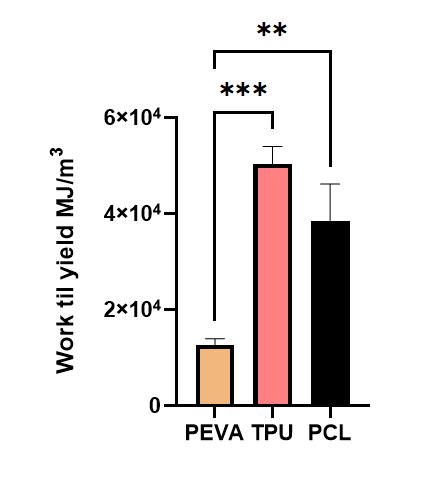

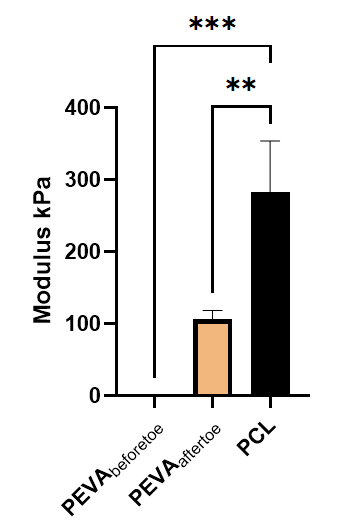


Figure S6. Statistical analysis of compressive properties.
